# Clinton W. Strang, D.D.S.

**Published:** 1922-02

**Authors:** 


### OBITUARY

Clinton W. Strang, D.D.S., died September 4, 1921, at his home in Bridgeport, Conn., in his 77th year. He graduated from the Pennsylvania College of Dental Surgery 1865, and practiced continuously for over fifty years in the city of Bridgeport, Conn. He was a modest man, but a man of fine personality and strong character. He was well known professionally, and he attracted everybody to him because of his genial and kindly character. He was one of those men who never appealed to the crowd, neither did he try by any means to make a name for himself. He was genuine and sincere, and he will be remembered for what he was and did. If our profession had more such men we should be better. We cherish his memory as one of the most satisfying experiences of our life. We extend our cordial sympathy to his widow and his family in their deep bereavement.

